# Sleep disturbance, anxiety, and depression in postoperative ovarian cancer patients: prevalence, associated factors, and symptom interrelationships

**DOI:** 10.3389/fonc.2026.1935380

**Published:** 2026-09-18

**Authors:** Ze Liu, Jialiang Yao, Jianhui Tian, Yun Yang, Yuan Yao, Huijuan Yang, Xi Cheng, Wei Jiang, Enzhao Cong, Yanhong Wang, Boer Shan

**Affiliations:** 1 Clinical Oncology Center, Shanghai Municipal Hospital of Traditional Chinese Medicine, Shanghai University of Traditional Chinese Medicine, Shanghai, China; 2 Institute of Oncology, Shanghai Municipal Hospital of Traditional Chinese Medicine, Shanghai University of Traditional Chinese Medicine, Shanghai, China; 3 Department of Gynecologic Oncology, Fudan University Shanghai Cancer Center, Shanghai, China; 4 Gynecologic Oncology, Obstetrics and Gynecology Hospital of Fudan University, Shanghai, China; 5 Shanghai Mental Health Center, Shanghai Jiao Tong University School of Medicine, Shanghai, China

**Keywords:** sleep disturbance, anxiety, depression, ovarian cancer, psycho-oncology, quality of life, symptom cluster

## Abstract

**Objective:**

This study aimed to investigate the prevalence, associated factors, and interrelationships of sleep disturbance, anxiety, and depression in postoperative ovarian cancer patients.

**Methods:**

A cross-sectional study was conducted among 302 postoperative patients with epithelial ovarian cancer, fallopian tube cancer, or primary peritoneal cancer. Sleep disturbance, anxiety, and depression were assessed using the Pittsburgh Sleep Quality Index (PSQI), Generalized Anxiety Disorder-7 (GAD-7), and Patient Health Questionnaire-9 (PHQ-9), respectively. Spearman correlation analyses were used to examine associations among symptoms. Binary logistic regression analyses identified factors independently associated with each symptom.

**Results:**

Among 302 postoperative ovarian cancer patients, sleep disturbance was the most prevalent psychological symptom, affecting 72.5% of participants, followed by depression (40.1%) and anxiety (26.5%). Co-occurrence of all three symptoms was observed in 18.2% of patients. In multivariable analyses, depressive symptoms were independently associated with sleep disturbance (OR = 1.33, 95% CI: 1.16–1.51), while disease recurrence (OR = 2.34, 95% CI: 1.15–4.77) and chemotherapy exposure were additionally associated with sleep disturbance. For anxiety symptoms, depressive symptoms remained independently associated with anxiety (OR = 1.37, 95% CI: 1.21–1.55), whereas urban residence was associated with lower odds of anxiety symptoms (OR = 0.49, 95% CI: 0.25–0.99). For depressive symptoms, anxiety symptoms (OR = 1.40, 95% CI: 1.24–1.58), sleep disturbance (OR = 1.16, 95% CI: 1.06–1.27), and cancer-related fatigue (OR = 1.45, 95% CI: 1.18–1.79) remained independent correlates. In addition, patients with average satisfaction with family life had higher odds of depressive symptoms compared with those reporting very high satisfaction (OR = 3.95, 95% CI: 1.41–11.03).

**Conclusions:**

Postoperative ovarian cancer patients experience a substantial burden of psychological symptoms, with sleep disturbance being the most prevalent. Distinct sociodemographic, clinical, and psychological factors were independently associated with sleep disturbance, anxiety, and depressive symptoms, which were also closely interrelated. Routine psychological screening and comprehensive supportive care integrating psychological, physical, and social interventions may help improve symptom management.

## Introduction

Ovarian cancer is the eighth most commonly diagnosed cancer and the eighth leading cause of cancer-related death among women worldwide (1). According to recent epidemiological estimates, there were approximately 324,398 new cases and 206,839 deaths globally in 2022 (2). Notable variations in the epidemiological profile of ovarian cancer exist across countries. In China, for example, an estimated 61,100 new cases and 32,600 deaths were reported in 2022 (3), accounting for a substantial proportion of the global burden and indicating a considerable disease burden at the national level.

In recent years, advances in comprehensive treatment strategies—including surgery, platinum-based chemotherapy, and the use of PARP inhibitors—have led to meaningful improvements in survival. Between 2000 and 2018, although the incidence of ovarian cancer in China showed an upward trend, mortality declined gradually (3). As a result, the population of ovarian cancer survivors has continued to expand. With the growing number of patients and prolonged survival, clinical attention has increasingly shifted toward reducing symptom burden and improving quality of life. Among the many factors affecting quality of life, the burden of psychological and neuropsychiatric symptoms represents an important yet still underrecognized aspect of care.

Psychological distress in patients with ovarian cancer is shaped by multiple interacting factors. First, disease-related characteristics play a key role (4):Owing to the insidious onset and lack of specific early symptoms, nearly 80% of patients are diagnosed at an advanced stage. Although the initial response rate to first-line treatment in advanced disease can reach approximately 80%, ovarian cancer is characterized by a high risk of recurrence and metastasis, with about 75% of patients experiencing relapse within two years. In addition, the overall five-year survival rate for advanced-stage disease remains relatively low. These challenges throughout the disease trajectory contribute to persistent uncertainty and psychological stress, thereby substantially increasing the mental health burden. Second, treatment modalities may also have a significant impact on psychological well-being. Surgical removal of reproductive organs can lead to abrupt hormonal changes, resulting in physiological and psychological symptoms similar to those observed during menopause (5). Adverse effects associated with chemotherapy further contribute to psychological distress, with patients commonly reporting a range of symptoms, including pain, sleep disturbance, anxiety, depression, and cognitive impairment (6–8). From a psychosocial perspective, the loss of reproductive organs may negatively affect self-identity and body image, and may even lead to social difficulties, thereby exacerbating psychological distress (9). In addition, cultural context plays an important role in shaping patients’ perceptions and experiences of illness. In some settings, cultural norms may reinforce stigma, which not only increases psychological burden but may also hinder timely expression and reporting of symptoms (10–12).

The impact of psychological distress in patients with ovarian cancer spans multiple dimensions and constitutes a range of clinically significant concerns. Persistent psychological burden may lead to sustained elevations in stress-related hormones, such as cortisol and catecholamines, which can suppress immune function and reduce immune surveillance against tumor cells, thereby potentially accelerating disease progression or recurrence (13–16). Psychological stress may also adversely affect treatment adherence. Patients experiencing high levels of stress are more likely to report treatment-related side effects or discontinue therapy due to psychological burden (17). Notably, emerging evidence suggests that chronic psychological stress may contribute to resistance to platinum-based chemotherapy and targeted therapies such as niraparib in ovarian cancer (18). In addition, psychological symptoms—including sleep disturbance, anxiety, and depression—often co-occur and interact with one another (19), forming what has been described as a psychological symptom cluster in ovarian cancer (20). Through synergistic effects, these symptoms amplify the overall burden and substantially impair quality of life across both physical and psychological domains. Taken together, these findings highlight that the identification and management of psychological symptoms are essential components of comprehensive ovarian cancer care, from both disease- and patient-centered perspectives.

Although psychological symptoms in patients with ovarian cancer have been widely investigated, previous studies have primarily focused on anxiety and depression rather than examining sleep disturbance alongside these symptoms (19, 21, 22). Studies simultaneously evaluating the coexistence and interrelationships of sleep disturbance, anxiety, and depression in ovarian cancer patients remain insufficient. In addition, some existing studies have included mixed gynecologic cancer populations (23, 24), with relatively limited attention given specifically to postoperative ovarian cancer patients, who may experience substantial physical and psychological stress following surgery. To address these gaps, this study aimed to systematically assess the prevalence, associated factors, and interrelationships of sleep disturbance, anxiety, and depression among postoperative ovarian cancer patients, thereby enhancing clinical recognition of these symptoms and informing more comprehensive management strategies.

The specific objectives of this study are as follows: (1) Assessment of the prevalence and severity of sleep disturbance, anxiety, and depression in patients with ovarian cancer; (2) Evaluation of the interrelationships among these symptoms; and (3) Identification of factors associated with these psychological symptoms.

## Methods

### Study design and setting

This cross-sectional study was conducted at two tertiary hospitals in Shanghai, China, and reported in accordance with the STROBE statement (25).

### Study population

Using convenience sampling, postoperative ovarian cancer patients attending outpatient clinics or admitted to wards at Shanghai Municipal Hospital of Traditional Chinese Medicine and Fudan University Shanghai Cancer Center were enrolled between December 2024 and December 2025.

### Inclusion criteria

1. Patients who had undergone comprehensive staging surgery or cytoreductive surgery for ovarian cancer;
2. In accordance with National Comprehensive Cancer Network (NCCN) guidelines (26), patients with a diagnosis of ovarian cancer, encompassing histopathologically confirmed epithelial ovarian cancer, fallopian tube cancer, or primary peritoneal cancer, were included.
3. Age ≥18 years, with the ability to understand and voluntarily provide written informed consent;
4. Adequate communication and cognitive ability to complete the questionnaire independently;
5. Complete clinical data available.

### Exclusion criteria

1. Non-epithelial ovarian malignancies, including ovarian germ cell tumors (e.g., immature teratoma), sex cord–stromal tumors (e.g., granulosa cell tumor, Sertoli–Leydig cell tumor), and other rare types;
2. A prior history of severe psychiatric disorders diagnosed before cancer (e.g., schizophrenia, bipolar disorder, or severe personality disorders);
3. Severe disturbances of consciousness, including delirium or coma.

### Sample size

Sample size was estimated using an empirical rule based on 23 candidate variables per outcome, applying a multiplier of 10 to yield a minimum of 230 participants (27). Accounting for an estimated 10% attrition rate, the target sample size was set at 256. A total of 318 patients were enrolled, of whom 302 met eligibility criteria and were included in the final analysis.

### Data collection and quality control

Data were collected via the Wenjuanxing online platform (www.wjx.cn) to ensure standardized data capture across both centers. The electronic questionnaire incorporated automated logic checks and completeness prompts, and all questionnaire items were set as mandatory fields, so incomplete questionnaires could not be submitted. Therefore, no missing values were present in the questionnaire dataset. Demographic and clinical variables were subsequently reviewed and verified by trained investigators through medical record review to ensure accuracy and completeness.

### Measurements

#### Demographics and clinical characteristics

Demographic and socioeconomic variables included residence, age (categorized into three groups) (3), body mass index (BMI; categorized according to Chinese criteria) (28), marital status, religious belief, employment status, education level, family life satisfaction(average, moderately satisfied, or very satisfied), monthly per capita household income, and insurance type. Clinical characteristics included menopausal status at surgery, International Federation of Gynecology and Obstetrics (FIGO) stage, histological type, number of chemotherapy cycles, use of poly(ADP-ribose) polymerase inhibitors (PARPi), receipt of traditional Chinese medicine (TCM), recurrence status, and family history of cancer.

### Measurement scales

Sleep disturbance was assessed using the Pittsburgh Sleep Quality Index (PSQI) (29), which has been validated in patients with cancer (30). In this study, the 18 self-rated items contributing to the scoring were included for analysis. These items generate seven component scores: subjective sleep quality, sleep latency, sleep duration, habitual sleep efficiency, sleep disturbances, use of sleep medication, and daytime dysfunction. Each component is scored on a 4-point scale (0–3), and the sum of the seven components yields a global PSQI score ranging from 0 to 21, with higher scores indicating poorer sleep quality. The total score was further categorized into four levels: good (0–5), fairly good (6–10), fairly poor (11–15), and poor (16–21) (31). Previous studies in oncology populations have suggested that a PSQI score ≥8 provides the optimal cutoff for identifying sleep disturbances, with a sensitivity of 90.4% and a specificity of 82.9% (32), Accordingly, a cutoff value of ≥8 was used in this study.

Anxiety symptoms were assessed using the Generalized Anxiety Disorder-7 (GAD-7). The scale consists of seven items, each rated on a 4-point scale from 0 (not at all) to 3 (nearly every day). The total score ranges from 0 to 21, with higher scores indicating greater severity of anxiety symptoms (33). Based on the total score, anxiety severity was categorized into four levels: none or minimal (0–4), mild (5–9), moderate (10–14), and severe (15–21) (33). A cutoff score of ≥7 was used in this study to indicate clinically significant anxiety symptoms (34).

Depressive symptoms were assessed using the Patient Health Questionnaire-9 (PHQ-9) (35). The scale comprises nine items, each rated on a 4-point scale from 0 (not at all) to 3 (nearly every day), reflecting symptom frequency over the past two weeks. The total score ranges from 0 to 27, with higher scores indicating greater severity of depressive symptoms (36). Based on the total score, depression severity was categorized into five levels: none (0–4), mild (5–9), moderate (10–14), moderately severe (15–19), and severe (20–27). A cutoff score of ≥7 was used in this study to indicate clinically significant depressive symptoms (37).

Pain intensity was assessed using the Numeric Rating Scale (NRS) (38). The NRS is a unidimensional, self-reported measure consisting of 11 integers ranging from 0 to 10, where 0 indicates no pain and 10 represents the worst imaginable pain. Participants were asked to select the number that best reflected their current level of pain on the electronic questionnaire. The selected value was recorded as the NRS score and used directly in subsequent analyses. In this study, an NRS score ≥4 was defined as the cutoff for clinically significant pain (39).

Functional status was assessed using the Karnofsky Performance Status scale (KPS) (40). The KPS score was determined by the treating physician based on the patient’s overall clinical condition at the time of assessment. Scores range from 0 to 100 in increments of 10, with 100 indicating normal functioning without evidence of disease and 0 indicating death.

Fatigue was assessed using the Revised Piper Fatigue Scale–Chinese Version (RPFS-CV), a Chinese version of the Revised Piper Fatigue Scale (41) that has been validated and widely used in clinical studies in China (42). The scale consists of 24 items. Item 1 determines whether the patient is experiencing fatigue; if so, the remaining items are completed. Item 2 records the duration of fatigue. Items 3–24 assess four dimensions of fatigue: behavioral/severity, affective meaning, sensory, and cognitive/mood. Each item is rated on an 11-point Likert scale (0–10). Based on the mean score, fatigue severity is categorized as none (0), mild (0–4), moderate [4–7), or severe [7–10] (43). If the item related to sexual activity is marked as “not applicable”, it is excluded from the calculation of both dimension scores and the total score; accordingly, it is also removed from the denominator when calculating mean values.

### Data management

Responses exported from Wenjuanxing were transferred into Microsoft Excel 2021 and cross-checked against original platform records, with discrepancies resolved using platform data as authoritative. Clinical variables were independently verified against medical records by two trained researchers. All personally identifiable information was removed to ensure ethical compliance.

### Statistical analysis

Statistical analyses were performed using R software (version 4.5.3).

Categorical variables were presented as frequencies and percentages (%). Continuous variables were assessed for normality using the Shapiro–Wilk test. Normally distributed data were presented as mean ± standard deviation (SD), whereas non-normally distributed data were reported as median (interquartile range, IQR). Participants were classified as having sleep disturbance, anxiety, or depression according to established clinical cutoff scores of the PSQI, GAD-7, and PHQ-9, respectively. Between-group comparisons for categorical variables were performed using the chi-square test, continuity-corrected chi-square test, or Fisher’s exact test, as appropriate. Continuous variables were compared using the independent-samples t-test or the Mann–Whitney U test. Correlations among variables were assessed using Spearman’s rank correlation. Univariate analyses were performed as exploratory analyses. Variables with clinical relevance or potential associations in univariate analyses (*P* < 0.10) were considered for inclusion in multivariable logistic regression models. Binary logistic regression analyses were performed to identify factors independently associated with sleep disturbance, anxiety, and depression. Sociodemographic and clinical variables identified from the univariate analyses were entered into the initial model. Sleep disturbance, anxiety, and depression were subsequently added in nested models. This hierarchical modeling strategy was used to evaluate whether the associations of individual psychological symptoms with each outcome remained after accounting for co-occurring psychological symptoms and to examine their relative contributions beyond sociodemographic and clinical factors.

## Results

A total of 318 postoperative ovarian cancer patients initially screened, 16 were excluded after applying eligibility criteria, yielding a final analytic sample of 302 participants (**Figure 1**).

**Figure 1:**
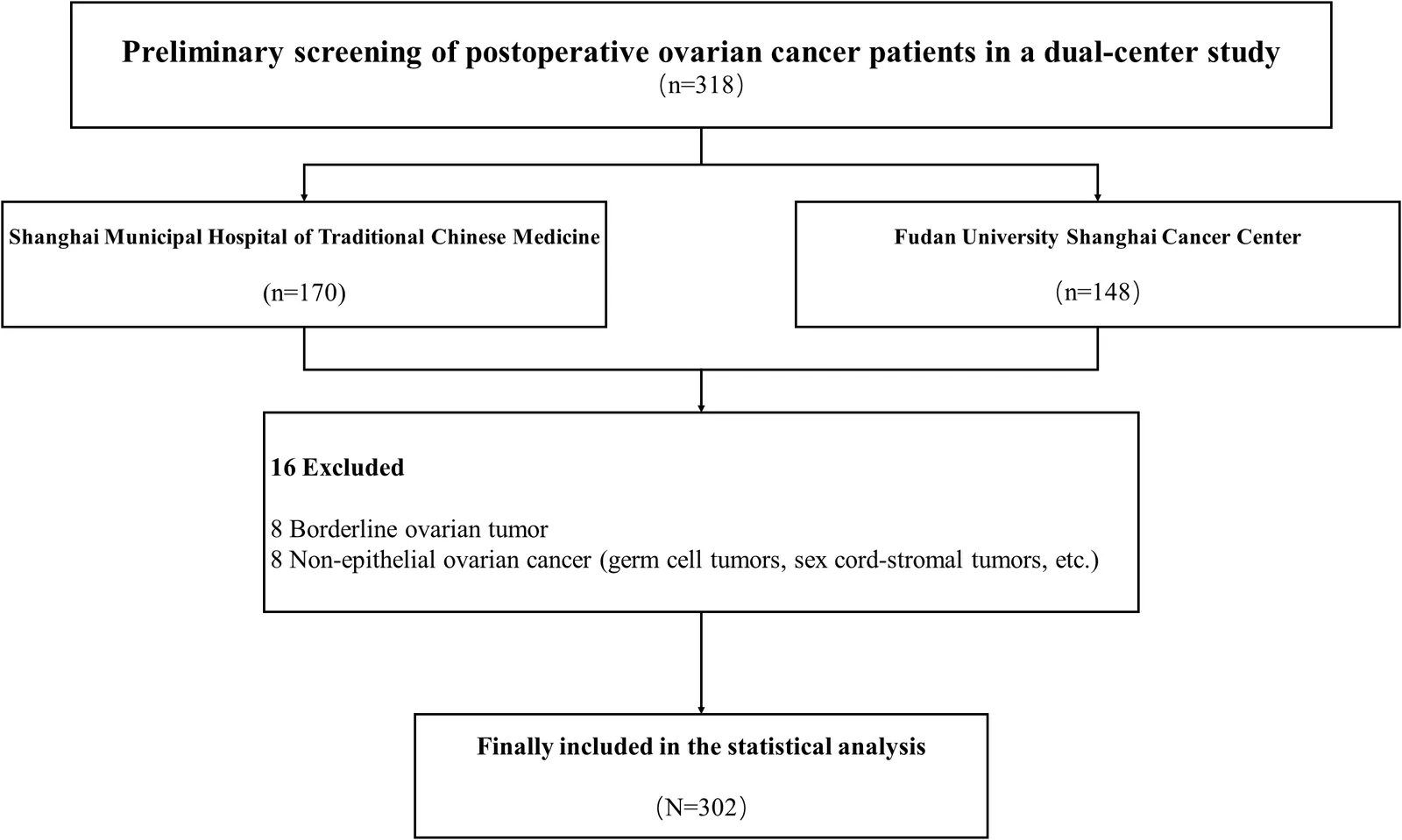
Flow diagram of patient selection. The figure illustrates the enrollment process of postoperative ovarian cancer patients in this dual-center cross-sectional study. Among 318 screened patients, 16 were excluded because of borderline ovarian tumors (*n* = 8) or non-epithelial ovarian cancer (*n* = 8). A total of 302 patients were included in the final statistical analysis.

### Participant characteristics

A total of 302 participants were enrolled, with a mean age of 54.8±9.4 years. Most participants were married, resided in urban areas, and had normal body weight. The majority were diagnosed with advanced-stage disease (FIGO stage III–IV), and high-grade serous carcinoma was the predominant histological subtype. More than half of the participants had received more than six chemotherapy cycles, and nearly half had experienced disease recurrence. Detailed demographic and clinical characteristics are presented in **Table 1**.

**Table 1:** Demographic, socioeconomic, and clinical characteristics of the study population (*N* = 302).

Variables	n (%)	Mean ± SD/median (IQR)
Residence		
Urban	177 (58.6)	
Town	66 (21.6)	
Rural	59 (19.5)	
**Age (years)**		54.8 ± 9.4
≤45	46 (15.2)	
46–55	119 (39.4)	
≥56	137 (45.4)	
**BMI (kg/m²)**		22.5 ± 3.1
Underweight (<18.5)	23 (7.6)	
Normal weight (18.5–23.9)	192 (63.6)	
Overweight (24.0–27.9)	72 (23.8)	
Obesity (≥28.0)	15 (5.0)	
Marital status		
Married	277 (91.7)	
Single/widowed/divorced	25 (8.3)	
Religious belief		
No	256 (84.8)	
Yes	46 (15.2)	
Employment status		
Employed	133 (44.0)	
Retired	136(45.0)	
Unemployed	33(11.0)	
Education level		
Primary school	48 (15.9)	
Junior high school	76 (25.2)	
High school/technical secondary school	71 (23.5)	
Junior college	61 (20.2)	
Bachelor’s degree or above	46 (15.2)	
Satisfaction with family life		
Very satisfied	89 (29.5)	
Moderately satisfied	148 (49.0)	
Average	65 (21.5)	
Monthly household income per capita (RMB)		
≤3,000	50 (16.6)	
3,001–5,000	87 (28.8)	
5,001–10,000	116 (38.4)	
>10000	49 (16.2)	
Medical insurance type		
Self-pay	10 (3.3)	
Rural medical insurance	33 (10.9)	
Urban resident medical insurance	86 (28.5)	
Urban employee medical insurance	173 (57.3)	
Menopausal status at surgery		
Yes	164 (54.3)	
No	138 (45.7)	
FIGO stage		
I	53 (17.5)	
II	29 (9.6)	
III	163 (54.0)	
IV	57 (18.9)	
Histological type		
High-grade serous carcinoma	236 (78.1)	
Low-grade serous carcinoma	4 (1.3)	
Endometrioid carcinoma	19 (6.3)	
Mucinous carcinoma	10 (3.3)	
Clear cell carcinoma	33 (10.9)	
Number of chemotherapy cycles		
0	15 (5.0)	
1-6	133 (44.0)	
>6	154 (51.0)	
Use of PARPi		
Yes	139 (46.0)	
No	163 (54.0)	
Use of TCM		
Yes	154 (51.0)	
No	148 (49.0)	
Recurrence		
Yes	145 (48.0)	
No	157 (52.0)	
Family history of cancer		
Yes	107 (35.4)	
No	195 (64.6)	
KPS		90 (80–100)
**NRS**		1 (0–2)
Reached clinical cutoff (≥4)		
YES	34 (11.3)	
NO	268 (88.7)	
RPFS-CV		
Mean score		2.8 (0–4.5)
Behavioral/severity dimension		2.2 (0–4)
Cognitive/mood dimension		2.7 (0–4.5)
Affective meaning dimension		2.3 (0–4.6)
Sensory dimension		3 (0–5.4)
Cancer-related fatigue (CRF)		
No CRF	94 (31.1)	
CRF present	208 (68.9)	
Mild	109 (36.1)	
Moderate	83 (27.5)	
Severe	16 (5.3)	

BMI, body mass index; FIGO, International Federation of Gynecology and Obstetrics; PARPi, poly(ADP-ribose) polymerase inhibitor; TCM, traditional Chinese medicine; KPS, Karnofsky Performance Status; NRS, Numerical Rating Scale; RPFS-CV, Revised Piper Fatigue Scale–Chinese Version; CRF, cancer-related fatigue; SD, standard deviation; IQR, interquartile range.

### Prevalence and severity of sleep disturbance, anxiety, and depression

The mean PSQI score was 10.6 ± 4.4, with sleep disturbance prevalent in 72.5% of participants; moderate-to-severe sleep disturbance accounted for 51.0%. Anxiety (GAD-7) and depression (PHQ-9) prevalence were 26.5% and 40.1%, respectively, with most patients presenting mild symptoms (median GAD-7: 4 [1–7]; median PHQ-9: 5 [3–8]). Co-occurrence of all three symptoms was observed in 18.2% of participants (**Table 2**; **Figure 2**).

**Table 2:** Prevalence and severity of sleep disturbance, anxiety, and depression (*N* = 302).

Measures	Mean ± SD/ median (IQR)	Severity	*n* (%)	Prevalence(%)
PSQI	10.6 ± 4.4	Good	42 (13.9)	72.5
		Fairly good	106 (35.1)	
		Fairly poor	109 (36.1)	
		Poor	45 (14.9)	
GAD-7	4 (1–7)	None/minimal anxiety	172 (57.0)	26.5
		Mild anxiety	99 (32.8)	
		Moderate anxiety	18 (6.0)	
		Severe anxiety	13 (4.3)	
PHQ-9	5 (3–8)	No depression	131 (43.4)	40.1
		Mild depression	126 (41.7)	
		Moderate depression	33 (10.9)	
		Moderately severe depression	6 (2.0)	
		Severe depression	6 (2.0)	
Sleep disturbance & Anxiety & Depression			55	18.2

PSQI, Pittsburgh Sleep Quality Index; GAD-7, Generalized Anxiety Disorder-7; PHQ-9, Patient Health Questionnaire-9.

**Figure 2:**
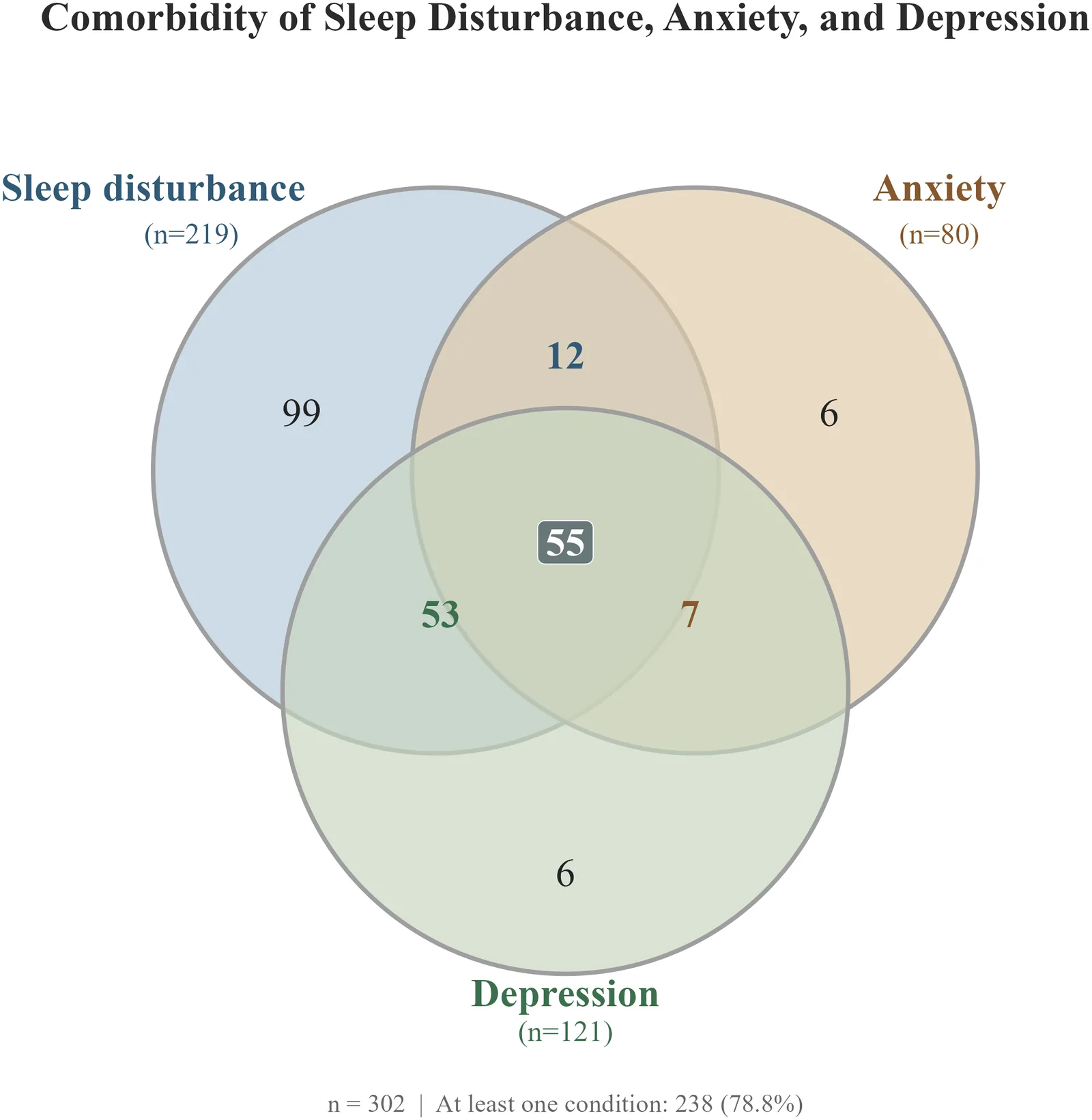
Venn diagram of co-occurrence among sleep disturbance, anxiety, and depression. The Venn diagram illustrates the overlap of the three symptoms in the study population (*N* = 302). sleep disturbance, anxiety symptoms, and depressive symptoms were present in 219 (72.5%), 80 (26.5%), and 121 (40.1%) patients, respectively. A total of 238 patients (78.8%) had at least one of the three symptoms. All three symptoms co-occurred in 55 patients (18.2%).

Stratified analyses revealed that sleep disturbance remained highly prevalent across all age groups and FIGO stage. Depression prevalence rose with both age and disease stage, while anxiety was relatively stable across age groups but trended downward with advancing stage. Across all subgroups, sleep disturbance was the most prevalent symptom, followed by depression and anxiety (**Figure 3**).

**Figure 3:**
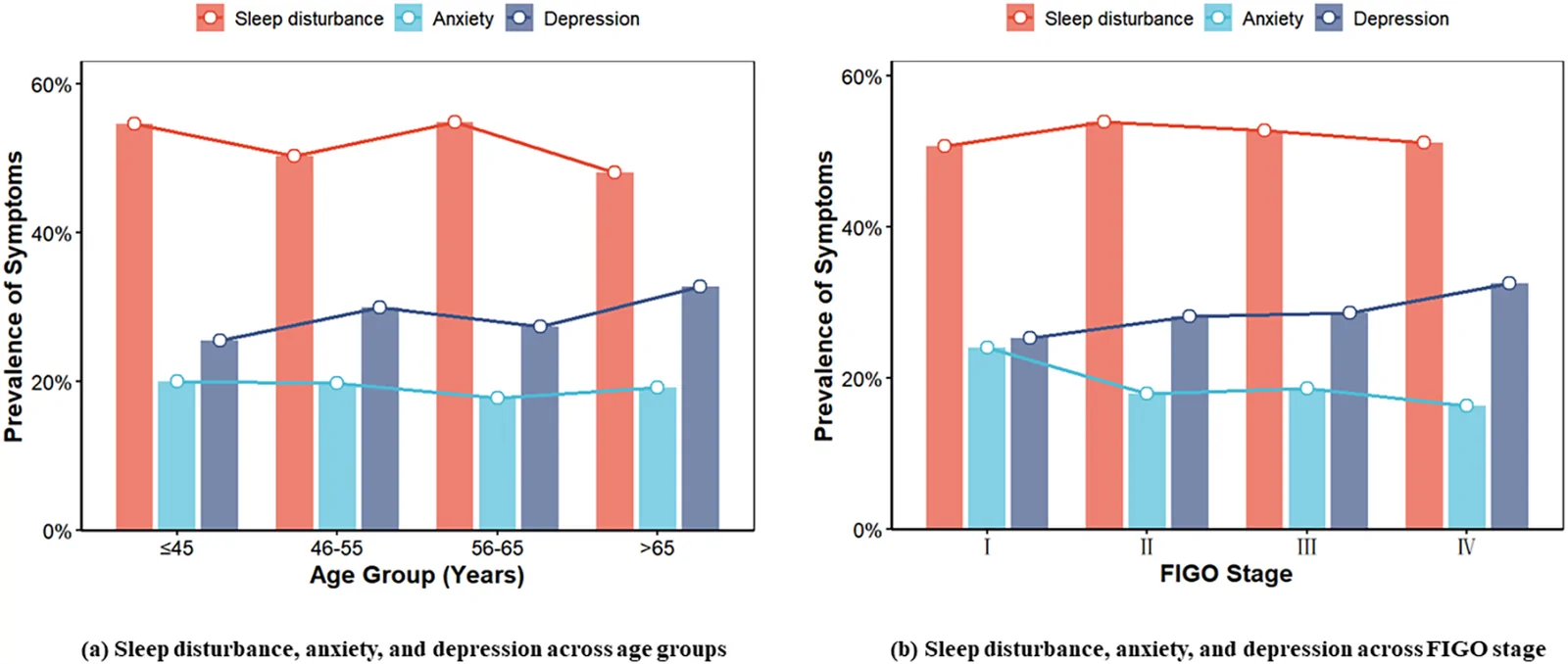
Prevalence of sleep disturbance, anxiety, and depressive symptoms according to age groups and FIGO stages among postoperative ovarian cancer patients. The prevalence of each symptom is shown across different age categories **(a)** and FIGO stages **(b)**. The prevalence of sleep disturbance remained consistently high across all subgroups, whereas depressive symptoms showed an increasing trend with older age and more advanced disease stage. Anxiety prevalence was relatively stable across age groups and showed a decreasing trend with advancing FIGO stage. Sleep disturbance was the most prevalent symptom across all subgroups, followed by depressive and anxiety symptoms. Symptom prevalence was calculated as the proportion of patients exceeding the predefined cutoff values for PSQI, GAD-7, and PHQ-9.

### Univariate analysis

Univariate analyses identified sleep disturbance as significantly associated with poorer functional status (KPS), higher pain intensity (NRS), greater fatigue (RPFS-CV), and higher anxiety and depression scores (all *P* < 0.05). Anxiety was significantly associated with residence, education level, monthly household income per capita, KPS, fatigue, sleep quality, and depression (all *P* < 0.05). Depression was associated with family life satisfaction, menopausal status at surgery, number of chemotherapy cycles, KPS, pain (NRS), fatigue, sleep quality, and anxiety (all *P* < 0.05). Variables not mentioned above did not show statistically significant associations with sleep disturbance, anxiety, or depression in univariable analyses (all *P*≥0.05) (**Table 3**, **4**).

**Table 3:** Univariate analysis of categorical variables among sleep disturbance, anxiety, and depression groups, n (%).

Variables	Total *N* = 302	Sleep disturbance n=219 (72.5)	Non- sleep disturbance n=83 (27.5)	*P*	Anxiety n=80 (26.5)	Non-anxiety n=222 (73.5)	*P*	Depression n=121(40.1)	Non-depression n=181 (59.9)	*P*
Demographic variables										
Residence				0.489			0.009**			0.350
Urban	177	131 (74.0)	46 (26.0)		37 (20.9)	140 (79.1)		67 (37.9)	110 (62.1)	
Non-urban	125	88 (70.4)	37 (29.6)		43 (34.4)	82 (65.6)		54 (43.2)	71 (56.8)	
Age (years)				0.270			0.881			0.35
≤45	46	30 (65.2)	16 (34.8)		11 (23.9)	35 (76.1)		14 (30.4)	32 (69.6)	
46–55	119	84 (70.6)	35 (29.4)		33 (27.7)	86 (72.3)		50 (42.0)	69 (58.0)	
≥56	137	105 (76.6)	32 (23.4)		36 (26.3)	101 (73.7)		57 (41.6)	80 (58.4)	
BMI (kg/m²)				0.537			0.204			0.129
Underweight	23	18 (78.3)	5 (21.7)		10 (43.5)	13 (56.5)		13 (56.5)	10 (43.5)	
Normal weight	192	142 (74.0)	50 (26.0)		50 (26.0)	142 (74.0)		79 (41.1)	113 (58.9)	
Overweight	72	50 (69.4)	22 (30.6)		18 (25.0)	54 (75.0)		26 (36.1)	46 (63.9)	
Obesity	15	9 (60.0)	6 (40.0)		2 (13.3)	13 (86.7)		3 (20.0)	12 (80.0)	
Marital status				0.684			0.858			0.994
Married	277	200 (72.2)	77 (27.8)		73 (26.4)	204 (73.6)		111 (40.1)	166 (59.9)	
Single/widowed/divorced	25	19 (76.0)	6 (24.0)		7 (28.0)	18 (72.0)		10 (40.0)	15 (60.0)	
Religious belief				0.898			0.768			0.427
No	256	186 (72.7)	70 (27.3)		67 (26.2)	189 (73.8)		105 (41.0)	151 (59.0)	
Yes	46	33 (71.7)	13 (28.3)		13 (28.3)	33 (71.7)		16 (34.8)	30 (65.2)	
Socioeconomic variables										
Employment status				0.879			0.258			0.304
Employed	133	95 (71.4)	38 (28.6)		39 (29.3)	94 (70.7)		58 (43.6)	75 (56.4)	
Retired	136	99 (72.8)	37 (27.2)		30 (22.1)	106 (77.9)		48 (35.3)	88 (64.7)	
Unemployed	33	25 (75.8)	8 (24.2)		11 (33.3)	22 (66.7)		15 (45.5)	18 (54.5)	
Education level				0.359			0.045^**^			0.053^*^
≤High school	195	138 (70.8)	57 (29.2)		59 (30.3)	136 (69.7)		86 (44.1)	109 (55.9)	
≥Junior college	107	81 (75.7)	26 (24.3)		21 (19.6)	86 (80.4)		35 (32.7)	72 (67.3)	
Satisfaction with family life				0.079^*^			0.099^*^			<0.001^**^
Very satisfied	89	58 (65.2)	31 (34.8)		21 (23.6)	68 (76.4)		24 (27.0)	65 (73.0)	
Moderately satisfied	148	108 (73.0)	40 (27.0)		35 (23.6)	113 (76.4)		58 (39.2)	90 (60.8)	
Average	65	53 (81.5)	12 (18.5)		24 (36.9)	41 (63.1)		39 (60.0)	26 (40.0)	
Monthly household income per capita (RMB)				0.928			0.043^**^			0.094^*^
≤5,000	137	99 (72.3)	38 (27.7)		44 (32.1)	93 (67.9)		62 (45.3)	75 (54.7)	
>5,000	165	120 (72.7)	45 (27.3)		36 (21.8)	129 (78.2)		59 (35.8)	106 (64.2)	
Medical insurance type				0.207			0.914			1.000
Self-pay	10	5 (50.0)	5 (50.0)		2 (20.0)	8 (80.0)		4 (40.0)	6 (60.0)	
Medical insurance	292	214(73.3)	78 (26.7)		78 (26.7)	214 (73.3)		117 (40.1)	175 (59.9)	
Clinical variables										
Menopausal status at surgery				0.189			0.503			0.015^**^
Yes	164	124 (75.6)	40 (24.4)		46 (28.0)	118 (72.0)		76 (46.3)	88 (53.7)	
No	138	95 (68.8)	43 (31.2)		34 (24.6)	104 (75.4)		45 (32.6)	93 (67.4)	
FIGO stage				0.893			0.336			0.421
I/II	82	59 (72.0)	23 (28.0)		25 (30.5)	57 (69.5)		30 (36.6)	52 (63.4)	
III/IV	220	160 (72.7)	60 (27.3)		55 (25.0)	165 (75.0)		91 (41.4)	129 (58.6)	
Histological type				0.562			0.632			0.328
High-grade serous carcinoma	236	173 (73.3)	63 (26.7)		61 (25.8)	175 (74.2)		98 (41.5)	138 (58.5)	
Non-high-grade serous carcinoma	66	46 (69.7)	20 (30.3)		19 (28.8)	47 (71.2)		23 (34.8)	43 (65.2)	
Chemotherapy cycles				0.093^*^			0.147			0.024^**^
0	15	10 (66.7)	5 (33.3)		7 (46.7)	8 (53.3)		8 (53.3)	7 (46.7)	
1-6	133	89 (66.9)	44 (33.1)		31 (23.3)	102 (76.7)		42 (31.6)	91 (68.4)	
>6	154	120 (77.9)	34 (22.1)		42 (27.3)	112 (72.7)		71 (46.1)	83 (53.9)	
Use of PARPi				0.569			0.207			0.085^*^
Yes	139	103 (74.1)	36 (25.9)		32 (23.0)	107 (77.0)		63 (45.3)	76 (54.7)	
No	163	116 (71.2)	47 (28.8)		48 (29.4)	115 (70.6)		58 (35.6)	105 (64.4)	
Use of TCM				0.549			0.322			0.526
Yes	154	114 (74.0)	40 (26.0)		37 (24.0)	117 (76.0)		59 (38.3)	95 (61.7)	
No	148	105 (70.9)	43 (29.1)		43 (29.1)	105 (70.9)		62 (41.9)	86 (58.1)	
Recurrence				0.284			0.713			0.495
Yes	145	101 (69.7)	44 (30.3)		37 (25.5)	108 (74.5)		61 (42.1)	84 (57.9)	
No	157	118 (75.2)	39 (24.8)		43 (27.4)	114 (72.6)		60 (38.2)	97 (61.8)	
Family history of cancer				0.132			0.652			0.342
Yes	107	72 (67.3)	35 (32.7)		30 (28.0)	77 (72.0)		39 (36.4)	68 (63.6)	
No	195	147 (75.4)	48 (24.6)		50 (25.6)	145 (74.4)		82 (42.1)	113 (57.9)	

* *P* < 0.1, ** *P* < 0.05.

**Table 4:** Univariate analysis of continuous variables among sleep disturbance, anxiety, and depression groups.

Variables	Mean ± SD/median (IQR)		*P*
	Yes	No	
Sleep disturbance			
KPS	90 (80–100)	90 (90–100)	0.004^**^
NRS	1 (0–2)	1 (0–2)	0.015^**^
RPFS-CV	3.3 (0.9–4.8)	0 (0–3.2)	<0.001^**^
GAD-7	4 (2–7)	3 (1–5)	0.008^**^
PHQ-9	6 (3–9)	3 (1–5)	<0.001^**^
Anxiety			
KPS	90 (70–97.5)	90 (80–100)	0.009^**^
NRS	1 (0–3)	1 (0–2)	0.054^*^
RPFS-CV	4.3 (2.5–6.2)	2.3 (0–4.0)	<0.001^**^
PSQI	12.4 ± 4.3	9.9 ± 4.3	<0.001^**^
PHQ-9	9 (7–11)	4 (2–7)	<0.001^**^
Depression			
KPS	90 (70–90)	90 (90–100)	<0.001^**^
NRS	2 (0.5–3)	1 (0–2)	<0.001^**^
RPFS-CV	4.3 (3.1–5.6)	0.7 (0–3.2)	<0.001^**^
PSQI	12.8 ± 4.1	9.1 ± 3.9	<0.001^**^
GAD7	7 (4–9)	2 (1–4)	<0.001^**^

*P < 0.1, ** *P* < 0.05.

### Multivariable analysis

Hierarchical binary logistic regression models were constructed separately for each outcome. Given the cross-sectional design of this study, the results of multivariable regression should be interpreted as statistical associations between variables rather than as independent risk factors or directional effects. Variables with *P* < 0.10 in univariable analyses were considered candidate variables for inclusion in multivariable models. Based on clinical knowledge and the events per variable (EPV) principle, additional adjustment variables were forced into each outcome-specific model to control for potential confounding and improve model stability. Multicollinearity was assessed using variance inflation factors (VIFs), and all variables had VIF values <5 (**Supplementary Table 1**), suggesting no evidence of substantial multicollinearity.

For sleep disturbance (**Table 5**), Model 1 showed that chemotherapy cycles, disease recurrence status, and RPFS-CV score were significantly associated with sleep disturbance. In Model 2a, after further adjustment for anxiety symptoms (GAD-7), chemotherapy cycles, disease recurrence status, and RPFS-CV score remained statistically significant, whereas GAD-7 score was not significantly associated with sleep disturbance. In Model 2b, after incorporating depressive symptoms (PHQ-9), PHQ-9 score emerged as a significant correlate of sleep disturbance (OR = 1.25, 95% CI: 1.12–1.41, *P* < 0.001), while the association between RPFS-CV score and sleep disturbance was attenuated and became statistically non-significant. In the final model (Model 3), after simultaneous adjustment for anxiety and depressive symptoms, PHQ-9 score remained significantly associated with sleep disturbance (OR = 1.33, 95% CI: 1.16–1.51, *P* < 0.001), whereas disease recurrence was independently associated with sleep disturbance (OR = 2.34, 95% CI: 1.15–4.77, *P* = 0.019). Chemotherapy cycles, analyzed as a categorical variable, were also significantly associated with sleep disturbance (*P* = 0.037). Compared with patients who had received more than six chemotherapy cycles, those who had received one to six cycles had lower odds of sleep disturbance (OR = 0.40, 95% CI: 0.19–0.84, *P* = 0.015). The final model showed good overall performance (likelihood ratio χ²=62.30, *P* < 0.001; Nagelkerke R²=0.270; Hosmer–Lemeshow test *P* = 0.811; area under the receiver operating characteristic curve (AUC)=0.779, 95% CI: 0.721–0.838).

**Table 5:** Hierarchical binary logistic regression analysis of factors associated with sleep disturbance.

Variables	Model 1			Model 2a (GAD-7)			Model 2b (PHQ-9)			Model 3		
	OR	95% CI	*P*	OR	95% CI	*P*	OR	95% CI	*P*	OR	95% CI	*P*
FIGO stage (I/II vs. III/IV)	1.12	0.58-2.15	0.733	1.10	0.57-2.11	0.782	1.10	0.56-2.16	0.776	1.20	0.60-2.38	0.611
Chemotherapy cycles			0.027**			0.028 **			0.043**			0.037**
0	0.31	0.07-1.30	0.109	0.31	0.07-1.29	0.107	0.29	0.07-1.25	0.097	0.28	0.06-1.20	0.087
1-6	0.39	0.19-0.79	0.009**	0.39	0.19-0.79	0.009 **	0.41	0.20-0.86	0.017**	0.40	0.19-0.84	0.015**
>6	Ref			Ref			Ref			Ref		
Satisfaction with family life			0.324			0.341			0.796			0.820
Average	1.82	0.81-4.11	0.149	1.80	0.79-4.06	0.160	1.31	0.56-3.07	0.537	1.28	0.54-3.01	0.578
Moderately satisfied	1.10	0.59-2.05	0.772	1.09	0.58-2.04	0.782	1.02	0.53-1.95	0.964	1.00	0.52-1.93	1.000
Very satisfied	Ref			Ref			Ref			Ref		
Use of PARPi (No vs. Yes)	1.05	0.58-1.92	0.862	1.04	0.57-1.90	0.906	1.06	0.57-1.98	0.848	1.13	0.60-2.13	0.702
Recurrence (No vs. Yes)	2.33	1.16-4.67	0.017**	2.32	1.16-4.64	0.018 **	2.25	1.11-4.56	0.025 **	2.34	1.15-4.77	0.019**
KPS	1.02	0.99-1.05	0.155	1.02	0.99-1.05	0.157	1.02	0.99-1.05	0.129	1.02	0.99-1.05	0.130
NRS	1.14	0.95-1.37	0.152	1.14	0.95-1.37	0.158	1.12	0.93-1.35	0.231	1.12	0.93-1.35	0.221
RPFS-CV	1.39	1.19-1.63	<0.001**	1.37	1.16-1.62	<0.001**	1.15	0.95-1.39	0.163	1.17	0.96-1.41	0.117
GAD-7	–	–	–	1.02	0.94-1.12	0.573	–	–	–	0.91	0.82-1.01	0.072
PHQ-9	–	–	–	–	–	–	1.25	1.12-1.41	<0.001**	1.33	1.16-1.51	<0.001**

OR, odds ratio; CI, confidence interval; Ref, reference indicator; HGSC, high-grade serous carcinoma.

Forced-included variables: FIGO stage, use of PARPi, recurrence.

** *P* < 0.05.

For anxiety symptoms (**Table 6**), Model 1 showed that residence and RPFS-CV score were significantly associated with anxiety symptoms. In Model 2a, after incorporating sleep quality (PSQI), urban residence and RPFS-CV score remained significantly associated with anxiety symptoms, whereas PSQI score was not significantly associated with anxiety symptoms. In Model 2b, after adding depressive symptoms (PHQ-9), PHQ-9 score was significantly associated with anxiety symptoms (OR = 1.36, 95% CI: 1.21–1.53, *P* < 0.001), while the association between RPFS-CV score and anxiety symptoms was attenuated and became statistically non-significant. In the final model (Model 3), after simultaneous adjustment for PSQI and PHQ-9 scores, PHQ-9 score remained independently associated with anxiety symptoms (OR = 1.37, 95% CI: 1.21–1.55, *P* < 0.001). In addition, urban residence remained associated with lower odds of anxiety symptoms (OR = 0.49, 95% CI: 0.25–0.99, *P* = 0.046). The final model showed good overall performance (likelihood ratio χ²=89.46, *P* < 0.001; Nagelkerke R²=0.374; Hosmer–Lemeshow test *P* = 0.501; AUC = 0.835, 95% CI: 0.785–0.885). To evaluate the potential confounding effects of unadjusted clinical variables, sensitivity analysis was performed by additionally including FIGO stage and treatment modality in the final model (**Supplementary Table 2**). The effect estimates and statistical inferences for depressive symptoms (PHQ-9) and fear of disease progression (RPFS-CV) remained stable. The effect size for residence was highly consistent between the primary and sensitivity analyses (OR = 0.49 vs. 0.50), although the *P* value slightly exceeded the conventional significance threshold after additional adjustment (*P* = 0.052). This change was likely attributable to increased standard errors due to the inclusion of additional parameters rather than a substantial alteration in the magnitude or direction of the association. Overall, the sensitivity analysis supported the robustness of the primary findings.

**Table 6:** Hierarchical binary logistic regression analysis of factors associated with anxiety.

Variables	Model 1			Model 2a (PSQI)			Model 2b (PHQ-9)			Model 3		
	OR	95% CI	*P*	OR	95% CI	*P*	OR	95% CI	*P*	OR	95% CI	*P*
Residence (Urban vs. not urban)	0.49	0.26-0.92	0.026^**^	0.48	0.25-0.91	0.026^**^	0.49	0.24-0.98	0.044^**^	0.49	0.25-0.99	0.046^**^
Education level (≤High school vs. ≥Junior college)	1.23	0.62-2.42	0.555	1.24	0.63-2.44	0.542	1.44	0.68-3.03	0.339	1.44	0.68-3.05	0.335
Satisfaction with family life			0.538			0.594			0.681			0.679
Average	1.09	0.49-2.45	0.831	1.06	0.47-2.39	0.897	0.86	0.35-2.08	0.731	0.86	0.35-2.09	0.736
Moderately satisfied	0.75	0.38-1.50	0.418	0.76	0.38-1.52	0.433	0.71	0.33-1.54	0.388	0.71	0.33-1.53	0.387
Very satisfied	Ref			Ref			Ref			Ref		
Monthly household income per capita (≤5,000 vs. >5,000)	1.04	0.53-2.03	0.915	1.09	0.55-2.14	0.808	0.81	0.38-1.73	0.591	0.80	0.38-1.72	0.576
KPS	1.02	0.99-1.04	0.215	1.01	0.99-1.04	0.252	1.03	1.00-1.06	0.090	1.03	1.00-1.06	0.088
NRS	1.06	0.91-1.23	0.471	1.05	0.90-1.23	0.522	1.06	0.90-1.26	0.473	1.06	0.90-1.26	0.469
RPFS-CV	1.51	1.30-1.76	<0.001^**^	1.45	1.23-1.70	<0.001^**^	1.13	0.93-1.37	0.217	1.13	0.93-1.37	0.209
PSQI	–	–	–	1.06	0.99-1.14	0.109	–	–	–	0.99	0.91-1.07	0.822
PHQ-9	–	–	–	–	–	–	1.36	1.21-1.53	<0.001^**^	1.37	1.21-1.55	<0.001^**^

** *P* < 0.05.

For depressive symptoms (**Table 7**), Model 1 showed that satisfaction with family life and RPFS-CV score were significantly associated with depressive symptoms. In Model 2a, after incorporating sleep quality (PSQI), satisfaction with family life and RPFS-CV score remained significantly associated with depressive symptoms, while PSQI scores were independently associated with depressive symptoms (OR = 1.18, 95% CI: 1.09–1.28, *P* < 0.001). In Model 2b, after adding anxiety symptoms (GAD-7), GAD-7 score was significantly associated with depressive symptoms (OR = 1.41, 95% CI: 1.26–1.58, *P* < 0.001), while family life satisfaction and RPFS-CV score remained statistically significant. In the final model (Model 3), after simultaneous adjustment for sleep quality and anxiety symptoms, GAD-7 score (OR = 1.40, 95% CI: 1.24–1.58, *P* < 0.001), PSQI score (OR = 1.16, 95% CI: 1.06–1.27, *P* = 0.001), RPFS-CV score (OR = 1.45, 95% CI: 1.18–1.79, *P* < 0.001), and satisfaction with family life (*P* = 0.019) remained independently associated with depressive symptoms. Additionally, compared with the very satisfied category, patients in the average category had higher odds of depressive symptoms (OR = 3.95, 95% CI: 1.41–11.03, *P* = 0.009). The final model showed good model performance (likelihood ratio χ²=179.51, *P* < 0.001; Nagelkerke R²=0.606; Hosmer–Lemeshow test *P* = 0.787; AUC = 0.907, 95% CI: 0.875–0.939).

**Table 7:** Hierarchical binary logistic regression analysis of factors associated with depression.

Variables	Model 1			Model 2a (PSQI)			Model 2b (GAD-7)			Model 3		
	OR	95% CI	*P*	OR	95% CI	*P*	OR	95% CI	*P*	OR	95% CI	*P*
Education level (≤High school vs. ≥Junior college)	1.29	0.66-2.53	0.462	1.28	0.64-2.56	0.492	1.59	0.74-3.44	0.238	1.45	0.67-3.16	0.350
Satisfaction with family life			0.021**			0.039**			0.012**			0.019**
Average	3.20	1.34-7.65	0.009**	2.97	1.19-7.40	0.019**	4.18	1.56-11.19	0.004**	3.95	1.41-11.03	0.009**
Moderately satisfied	1.22	0.60-2.50	0.583	1.14	0.54-2.41	0.727	1.32	0.59-2.97	0.497	1.22	0.53-2.82	0.639
Very satisfied	Ref			Ref			Ref			Ref		
Monthly household income per capita (≤5,000 vs. >5,000)	0.96	0.48-1.89	0.896	1.10	0.54-2.25	0.794	0.56	0.25-1.25	0.159	0.67	0.30-1.53	0.346
Menopausal status at surgery (No vs. Yes)	0.76	0.41-1.39	0.370	0.79	0.42-1.48	0.459	0.62	0.31-1.23	0.168	0.63	0.31-1.28	0.198
Chemotherapy cycles			0.528			0.441			0.727			0.602
0	1.74	0.36-8.37	0.490	2.64	0.47-14.65	0.268	1.82	0.29-11.56	0.528	2.70	0.36-20.24	0.334
1-6	0.79	0.39-1.61	0.519	0.90	0.43-1.88	0.774	0.90	0.41-1.96	0.785	1.00	0.45-2.26	0.992
>6	Ref			Ref			Ref			Ref		
Use of PARPi (No vs. Yes)	0.68	0.36-1.29	0.241	0.65	0.33-1.28	0.216	0.51	0.25-1.06	0.071	0.51	0.24-1.08	0.077
Recurrence (No vs. Yes)	0.96	0.48-1.93	0.915	0.76	0.36-1.58	0.460	0.93	0.43-2.02	0.852	0.75	0.33-1.69	0.482
FIGO stage (I/II vs. III/IV)	1.10	0.54-2.24	0.798	1.17	0.55-2.47	0.685	0.73	0.33-1.62	0.441	0.78	0.34-1.80	0.560
KPS	1.00	0.97-1.02	0.781	0.99	0.97-1.02	0.621	0.99	0.96-1.02	0.380	0.99	0.96-1.02	0.371
NRS	1.02	0.85-1.21	0.857	1.00	0.83-1.20	0.995	0.98	0.80-1.19	0.821	0.96	0.78-1.17	0.668
RPFS-CV	1.79	1.51-2.12	<0.001**	1.65	1.38-1.98	<0.001**	1.56	1.28-1.89	<0.001**	1.45	1.18-1.79	<0.001**
PSQI	–	–	–	1.18	1.09-1.28	<0.001**	–	–	–	1.16	1.06-1.27	0.001**
GAD7	–	–	–	–	–	–	1.41	1.26-1.58	<0.001**	1.40	1.24-1.58	<0.001**

Forced-included variables: FIGO stage, recurrence.

** *P* < 0.05.

## Discussion

### High overall burden: sleep disturbance as the most prominent feature

For the three symptom domains, this study did not use the conventional epidemiological cutoffs (PSQI >5, GAD-7 ≥10, and PHQ-9 ≥10). Instead, considering the characteristics of cancer survivors and referring to previous studies evaluating the applicability of these instruments in cancer populations, we adopted cutoffs considered more appropriate for cancer patients to facilitate more targeted screening of sleep disturbance, anxiety, and depressive symptoms. Among 302 postoperative ovarian cancer patients, the prevalence rates of sleep disturbance, anxiety, and depressive symptoms were 72.5%, 26.5%, and 40.1%, respectively, with sleep disturbance being the most prevalent symptom.

A relatively stringent PSQI cutoff (≥8) was adopted based on a study conducted in Taiwan (32), with consideration given to both the characteristics of cancer populations and the relatively similar cultural background. This higher threshold may have resulted in a lower estimated prevalence of sleep disturbance than that obtained using the conventional cutoff. Nevertheless, the prevalence in our study remained substantially higher than that reported in Western populations (59–60.7%) (44, 45), and was comparable to that reported in a previous Taiwanese study (75.4–80.0%) (46). Similarly, a study of 195 patients with gynecological cancers in mainland China reported that 77.43% had poor sleep quality (47). Collectively, these findings suggest that poor sleep is common among Chinese patients with cancer.

The cutoff of GAD-7 ≥7 used in this study was derived from a study of 2,141 patients with cancer (34), in which this threshold achieved the optimal balance between sensitivity and specificity compared with the commonly recommended cutoff of ≥10. Although the use of this lower cutoff may result in a higher estimated prevalence of anxiety symptoms, the prevalence observed in our study (26.5%) was lower than that reported in a previous meta-analysis (33%) (48) and a systematic review (37%) (49). A previous study of Chinese patients with ovarian cancer reported a higher prevalence of anxiety symptoms (51.5%) (19). However, that study focused on hospitalized patients, who may experience greater anxiety related to disease severity and treatment intensity, and used the Hospital Anxiety and Depression Scale (HADS) rather than the GAD-7, which may also contribute to differences in prevalence estimates.

The cutoff of PHQ-9 ≥7 used in this study was derived from a study of 2,141 patients with cancer (37), which found that a cutoff of ≥7 provided the optimal screening performance, with a sensitivity of 83% (95% CI, 78%–89%) and specificity of 61% (95% CI, 59%–63%). The use of this cutoff may provide greater clinical utility for screening depressive symptoms in patients with cancer. However, because it is lower than the conventional cutoff of ≥10, it may result in a higher estimated prevalence, which should be considered when comparing our findings with other studies using the PHQ-9. The prevalence of depressive symptoms in our study was closely aligned with that reported in a large cohort study (40%) (50) and was also comparable to that reported in a Chinese study (47%) (19), but was higher than the estimate from a systematic review (27%) (48). In contrast, a study of patients with ovarian cancer in Australia reported a substantially lower prevalence of depressive symptoms (14.7%) using the HADS (51), including 5.9% with clinical depression and 8.8% with subclinical depression.

Overall, the prevalence of sleep disturbance, anxiety, and depressive symptoms differed across studies, potentially reflecting differences in disease status, treatment burden, cultural context, and assessment methods. Given the use of population-specific cutoffs in the present study, caution is warranted when comparing our findings with those from other studies.

### Associated factor interpretation

Regarding sleep disturbance, depressive symptoms showed the strongest and most consistent association. After adjustment for anxiety symptoms and other clinical variables, PHQ-9 scores remained independently associated with sleep disturbance, whereas anxiety symptoms were no longer statistically significant. Specifically, each one-point increase in PHQ-9 score was associated with a 33% increase in the odds of sleep disturbance (OR = 1.33, 95% CI: 1.16–1.51), suggesting a clinically meaningful association between depressive symptom severity and sleep disturbance. This finding differs from a previous cross-sectional study of ovarian cancer patients in Australia, which reported a significant association between anxiety symptoms and sleep disturbance (52). The discrepancy may reflect differences in study populations, cultural backgrounds, healthcare contexts, or other unmeasured factors across regions. Disease recurrence was also independently associated with sleep disturbance. Patients with recurrent disease had more than two-fold higher odds of sleep disturbance compared with those without recurrence (OR = 2.34, 95% CI: 1.15–4.77), suggesting that recurrent disease was associated with a greater burden of sleep disturbance. Previous cohort studies have also explored the relationship between sleep disturbance and cancer outcomes. For example, a cohort study among patients with completely platinum-sensitive ovarian cancer in Canada reported an association between sleep disturbance and recurrence risk (53), and a previous Chinese cohort study suggested a similar relationship (54). However, these longitudinal findings and our cross-sectional results represent different temporal perspectives, and the directionality of the association between sleep disturbance and disease recurrence remains unclear. Chemotherapy exposure was additionally associated with sleep disturbance, with patients receiving 1–6 chemotherapy cycles having lower odds of sleep disturbance than those receiving more than six cycles (OR = 0.40, 95% CI: 0.19–0.84). This finding suggests that greater chemotherapy exposure was associated with a higher burden of sleep disturbance. Consistent with our findings, a cross-sectional study conducted in China among patients with gynecological cancers also reported an association between increased sleep disturbance severity and a greater number of chemotherapy cycles (47). Given the cross-sectional design of the present study, the temporal relationship between chemotherapy exposure and sleep disturbance cannot be determined, and these associations should therefore be interpreted cautiously.

For anxiety symptoms, depressive symptoms were the strongest independent correlate. After adjustment for depressive symptoms, the association between sleep quality and anxiety was no longer significant. Notably, each one-point increase in PHQ-9 score was associated with a 37% increase in the odds of anxiety symptoms (OR = 1.37, 95% CI: 1.21–1.55), indicating a clinically meaningful association between depressive symptom burden and anxiety symptoms. Urban residence was associated with lower odds of anxiety symptoms (OR = 0.49, 95% CI: 0.25–0.99). A prospective study from the United States investigating psychosocial functioning among patients with epithelial ovarian cancer showed that urban patients experienced greater improvement in psychological distress during the first year after diagnosis and had significantly lower overall distress levels at one-year follow-up compared with rural patients (55). These findings are consistent with the trend observed in the present study, in which urban residence was associated with a lower likelihood of anxiety symptoms, suggesting that residential environment and related social resources may be associated with psychological well-being among patients with ovarian cancer.

For depressive symptoms, anxiety symptoms and poor sleep quality remained significant correlates, reflecting the substantial overlap within the psychological symptom cluster. Notably, each one-point increase in GAD-7 score was associated with a 40% increase in the odds of depressive symptoms (OR = 1.40, 95% CI: 1.24–1.58), while each one-point increase in PSQI score was associated with a 16% increase in the odds of depressive symptoms (OR = 1.16, 95% CI: 1.06–1.27). These findings indicate that anxiety and sleep disturbance were not only statistically associated with depressive symptoms but also showed clinically meaningful relationships within this co-occurring symptom cluster. Beyond these co-occurring psychological symptoms, cancer-related fatigue (CRF) and family-related factors showed independent associations with depressive symptoms. Each one-point increase in RPFS-CV score was associated with a 45% increase in the odds of depressive symptoms (OR = 1.45, 95% CI: 1.18–1.79), suggesting that greater fatigue burden was clinically relevant to depressive symptom severity. Although CRF may overlap with emotional distress and sleep disturbance, its independent association with depressive symptoms highlights the close coexistence of physical and psychological symptom burden in patients with ovarian cancer. Persistent fatigue may impair daily functioning, reduce physical activity, and restrict social participation, whereas depressive symptoms may also intensify the perception and impact of fatigue (56, 57). In addition, compared with patients who were very satisfied with family life, those with average satisfaction with family life had nearly four-fold higher odds of depressive symptoms (OR = 3.95, 95% CI: 1.41–11.03). This association highlights the potential relevance of family-related psychosocial factors in psychological outcomes after ovarian cancer treatment. Consistent with our findings, a Chinese study reported a negative association between depressive symptoms and family resilience (58), while a mixed cross-sectional study from Finland also found that family-related factors were closely associated with psychological well-being among patients with gynecological cancers (59). Greater family-related psychosocial resources may be associated with better psychological adaptation by providing emotional reassurance and practical assistance. However, satisfaction with family life was assessed using a single-item measure rather than a standardized multi-item scale, which may not fully capture the complexity of family-related psychosocial experiences. Future studies incorporating validated structured instruments are warranted to provide a more comprehensive and precise assessment of the role of family-related psychosocial factors in psychological outcomes.

### Interrelationships among sleep disturbance, anxiety, and depressive symptoms

Sleep disturbance, anxiety, and depressive symptoms frequently co-occur in patients (60). Similarly, our study found that these symptoms were also significantly correlated to varying degrees among postoperative ovarian cancer patients (**Supplementary Table 3**), while several associations remained statistically significant after adjustment for relevant covariates in multivariable regression analyses. However, these associations should be interpreted cautiously because the PSQI, GAD-7, and PHQ-9 assess closely related constructs, with some potential measurement overlap. In particular, the PHQ-9 includes an item assessing sleep disturbances, which may partially contribute to the observed association between sleep disturbance and depressive symptoms. Nevertheless, the observed co-occurrence suggests that sleep disturbance, anxiety, and depressive symptoms may constitute an interconnected symptom cluster rather than occur in isolation, highlighting the potential value of comprehensive assessment of these symptoms in postoperative ovarian cancer patients. Given the cross-sectional design, however, the observed associations should not be interpreted as evidence of temporal or causal relationships among these symptoms.

### Clinical implications

Several considerations for clinical practice emerge from these findings. First, routine screening for psychological symptoms using standardized assessment tools could be strengthened. In routine oncology practice, clinical attention is often directed primarily toward disease control and antitumor treatment, while psychological symptoms may receive less attention. Reliance solely on symptom-driven inquiries may fail to identify patients experiencing psychological distress, particularly when symptoms are underreported or overlooked. In the present study, standardized patient-reported outcome measures identified a considerable prevalence of psychological symptoms, especially sleep disturbance, highlighting the potential value of incorporating routine psychological screening into comprehensive cancer care. Second, symptom-specific considerations may facilitate more targeted supportive care. For sleep disturbance, enhanced monitoring may be warranted for patients with recurrent disease and those undergoing multiple chemotherapy cycles, who may experience greater disease- and treatment-related burden. For anxiety symptoms, greater attention may be warranted for patients living in non-urban areas, who may have limited access to cancer-related education, health information, and psychosocial support resources. For depressive symptoms, patients experiencing greater CRF may warrant closer attention. Assessment of depressive symptoms may also benefit from considering patients’ family circumstances, particularly among those reporting lower satisfaction with family life. Third, these findings support the potential value of a symptom-cluster approach to psychological care in postoperative ovarian cancer patients. Because sleep disturbance, anxiety, and depressive symptoms frequently co-occur, routine psychological assessment that encompasses all three symptoms may facilitate early identification and comprehensive management.

### Limitations

This study has several limitations. First, because of the cross-sectional design, causal relationships between the identified factors and sleep disturbance, anxiety, and depressive symptoms, as well as among these symptoms, cannot be inferred from our findings. Second, participants were recruited using convenience sampling from two tertiary hospitals in Shanghai, China. Therefore, the findings may not be generalizable to patients treated in community or non-tertiary healthcare settings, or in other regions. Third, although validated self-report instruments were used to assess sleep disturbance, anxiety, and depressive symptoms, these measures were not equivalent to structured psychiatric interviews and may therefore be subject to reporting bias, common-method bias, and symptom misclassification. Fourth, although multiple sociodemographic and clinical variables were adjusted for, residual confounding from unmeasured factors, such as fear of cancer recurrence, perceived social support, and previous or ongoing psychological treatment, cannot be excluded. In addition, the time since surgery was not systematically collected. Patients assessed shortly after surgery may differ substantially from long-term survivors in their psychological status, potentially influencing the observed associations between sleep disturbance, anxiety, and depressive symptoms and relevant clinical or psychosocial factors and introducing residual confounding. Finally, the study included only patients with epithelial ovarian cancer, fallopian tube cancer, and primary peritoneal cancer, limiting the applicability of the findings to other ovarian cancer subtypes.

## Conclusion

Postoperative ovarian cancer patients experience a substantial burden of psychological symptoms, with sleep disturbance being the most prevalent symptom. Distinct sociodemographic, clinical, and psychological factors were independently associated with sleep disturbance, anxiety, and depressive symptoms, highlighting the multifactorial nature of psychological distress in this population. These symptoms were also closely interrelated and frequently co-occurred. These findings highlight the potential value of routine psychological screening and comprehensive supportive care that considers psychological, physical, and social factors. Further longitudinal studies are warranted to clarify the temporal relationships among these symptoms and inform more effective symptom management strategies.

## Data Availability

Data are available upon request to the corresponding authors, subject to Institutional Review Board approval of the Shanghai Municipal Hospital of Traditional Chinese Medicine. Requests to access the datasets should be directed to tumorwork996@126.com.
