# Supplementary material for: Sleep disturbance, anxiety, and depression in postoperative ovarian cancer patients: prevalence, associated factors, and symptom interrelationships
Source: Front Oncol. 2026 Sep 18;16:1935380. doi: 10.3389/fonc.2026.1935380 (PMC13630616; doi:10.3389/fonc.2026.1935380)
Supplement: Supplementary file 1 [file Table1.docx]

**Supplementary Table S1. Assessment of multicollinearity among variables included in multivariable logistic regression models**

| **Variables** | **Tolerance** | **VIF** |
| --- | --- | --- |
| **Insomnia** |  |  |
| FIGO stage (I/II vs. III/IV) | 0.875 | 1.143 |
| Chemotherapy cycles |  |  |
| 0 | 0.812 | 1.231 |
| 1-6 | 0.632 | 1.583 |
| >6 | **Ref** |  |
| Satisfaction with family life |  |  |
| Average | 0.692 | 1.446 |
| Moderately satisfied | 0.702 | 1.424 |
| Very satisfied | **Ref** |  |
| Use of PARPi (No vs. Yes) | 0.808 | 1.237 |
| Recurrence (No vs. Yes) | 0.686 | 1.458 |
| KPS | 0.606 | 1.650 |
| NRS | 0.772 | 1.296 |
| RPFS-CV | 0.422 | 2.370 |
| GAD-7 | 0.505 | 1.981 |
| PHQ-9 | 0.378 | 2.647 |
| **Anxiety** |  |  |
| Residence (Urban vs. not urban) | 0.774 | 1.291 |
| Education level  (≤High school vs. ≥Junior college) | 0.804 | 1.243 |
| Satisfaction with family life |  |  |
| Average | 0.644 | 1.553 |
| Moderately satisfied | 0.694 | 1.442 |
| Very satisfied | **Ref** |  |
| Monthly household income per capita  (≤5,000 vs. >5,000) | 0.683 | 1.465 |
| KPS | 0.605 | 1.653 |
| NRS | 0.759 | 1.318 |
| RPFS-CV | 0.412 | 2.425 |
| PSQI | 0.668 | 1.497 |
| PHQ-9 | 0.442 | 2.263 |
| FIGO stage | 0.868 | 1.152 |
| Chemotherapy cycles |  |  |
| 0 | 0.797 | 1.255 |
| 1-6 | 0.624 | 1.603 |
| >6 | **Ref** |  |
| Use of PARPi (No vs. Yes) | 0.814 | 1.229 |
| Recurrence (No vs. Yes) | 0.661 | 1.513 |
| **Depression** |  |  |
| Education level  (≤High school vs. ≥Junior college) | 0.809 | 1.237 |
| Satisfaction with family life |  |  |
| Average | 0.657 | 1.523 |
| Moderately satisfied | 0.692 | 1.445 |
| Very satisfied | **Ref** |  |
| Monthly household income per capita  (≤5,000 vs. >5,000) | 0.742 | 1.347 |
| Menopausal status at surgery (No vs. Yes) | 0.903 | 1.108 |
| Chemotherapy cycles |  |  |
| 0 | 0.801 | 1.248 |
| 1-6 | 0.623 | 1.606 |
| >6 | **Ref** |  |
| Use of PARPi (No vs. Yes) | 0.795 | 1.257 |
| Recurrence (No vs. Yes) | 0.659 | 1.517 |
| FIGO stage (I/II vs. III/IV) | 0.862 | 1.160 |
| KPS | 0.587 | 1.703 |
| NRS | 0.760 | 1.316 |
| RPFS-CV | 0.460 | 2.174 |
| PSQI | 0.737 | 1.358 |
| GAD7 | 0.650 | 1.538 |

**Supplementary Table S2. Sensitivity Analysis: Additional Adjustment for FIGO Stage, Chemotherapy Cycles, PARPi Use, and Recurrence in the Anxiety Model**

| **Variables** | **Final Model (EPV=8.0)** | | | **Sensitivity Analysis (EPV=5.3)** | | |
| --- | --- | --- | --- | --- | --- | --- |
|  | **OR** | **95% CI** | ***P*** | **OR** | **95% CI** | ***P*** |
| Residence (Urban vs. not urban) | 0.49 | 0.25-0.99 | 0.046^**^ | 0.50 | 0.25-1.01 | 0.052 |
| Education level  (≤High school vs. ≥Junior college) | 1.44 | 0.68-3.05 | 0.335 | 1.45 | 0.68-3.09 | 0.339 |
| Satisfaction with family life |  |  | 0.679 |  |  | 0.757 |
| Average | 0.86 | 0.35-2.09 | 0.736 | 0.88 | 0.35-2.19 | 0.783 |
| Moderately satisfied | 0.71 | 0.33-1.53 | 0.387 | 0.75 | 0.34-1.63 | 0.465 |
| Very satisfied | Ref |  |  | Ref |  |  |
| Monthly household income per capita  (≤5,000 vs. >5,000) | 0.80 | 0.38-1.72 | 0.576 | 0.79 | 0.36-1.74 | 0.561 |
| KPS | 1.03 | 1.00-1.06 | 0.088 | 1.03 | 1.00-1.06 | 0.090 |
| NRS | 1.06 | 0.90-1.26 | 0.469 | 1.05 | 0.88-1.26 | 0.551 |
| RPFS-CV | 1.13 | 0.93-1.37 | 0.209 | 1.14 | 0.94-1.39 | 0.194 |
| PSQI | 0.99 | 0.91-1.07 | 0.822 | 1.00 | 0.92-1.08 | 0.920 |
| PHQ-9 | 1.37 | 1.21-1.55 | <0.001^**^ | 1.37 | 1.21-1.55 | <0.001^**^ |
| FIGO stage | - | - | - | 1.39 | 0.68-2.87 | 0.368 |
| Chemotherapy cycles | - | - | - | Ref |  | 0.462 |
| 0 | - | - | - | 2.23 | 0.53-9.43 | 0.277 |
| 1-6 | - | - | - | 0.94 | 0.43-2.06 | 0.873 |
| >6 | - | - | - | Ref |  |  |
| Use of PARPi (No vs. Yes) | - | - | - | 1.25 | 0.62-2.51 | 0.535 |
| Recurrence (No vs. Yes) | - | - | - | 1.01 | 0.47-2.20 | 0.974 |

 EPV, Events Per Variable

Forced-included variables: FIGO stage, chemotherapy cycles, use of PARPi, recurrence

**Notes: The final anxiety model included 10 parameters with 80 events (EPV=8.0). The sensitivity analysis model additionally included FIGO stage, chemotherapy cycles, PARPi use, and recurrence status, totaling 15 parameters with 80 events (EPV=5.3). Core findings were robust: (1) PHQ-9 remained a strong independent risk factor for anxiety (OR=1.37, 95% CI: 1.21–1.55, P<0.001 in both models). (2) The effect size for Residence was virtually unchanged (OR=0.50 vs. 0.493), with the *P*-value marginally crossing 0.05 (*P*=0.052 vs. 0.046) attributable to standard error inflation from the increased number of parameters. No core finding was altered in direction or substantially in magnitude. All additionally adjusted clinical variables were non-significant (all *P*>0.05).**

**Correlation analysis**

Spearman rank correlation analysis of PSQI, GAD-7, PHQ-9, NRS, KPS, and RPFS-CV revealed significant inter-variable correlations (all *P*<0.05), with coefficients ranging from 0.2 to 0.658. All variables were therefore retained in subsequent multivariable models (Table 5; Figure 4).

**Supplementary Table S3. Correlation Analysis of PSQI, GAD-7, PHQ-9, NRS, KPS, and RPFS-CV**

| **Variables** | **PSQI** | **GAD-7** | **PHQ-9** | **NRS** | **KPS** | **RPFS-CV** |
| --- | --- | --- | --- | --- | --- | --- |
| PSQI | 1.000 | 0.281^**^ | 0.529^**^ | 0.200^**^ | -0.248^**^ | 0.433^**^ |
| GAD-7 | - | 1.000 | 0.628^**^ | 0.202^**^ | -0.274^**^ | 0.488^**^ |
| PHQ-9 | - | - | 1.000 | 0.290^**^ | -0.477^**^ | 0.658^**^ |
| NRS | - | - | - | 1.000 | -0.366^**^ | 0.324^**^ |
| KPS | - | - | - | - | 1.000 | -0.592^**^ |
| RPFS-CV | - | - | - | - | - | 1.000 |

*** P* < 0.05
